# Association between Sleep Quality and Mental Health among Patients at a Post-COVID-19 Recovery Clinic

**DOI:** 10.3390/brainsci12050586

**Published:** 2022-04-30

**Authors:** Sara Nowakowski, Manasa Kokonda, Rizwana Sultana, Brittany B. Duong, Sarah E. Nagy, Mohammed F. Zaidan, Mirza M. Baig, Bryan V. Grigg, Justin Seashore, Rachel R. Deer

**Affiliations:** 1 Department of Medicine, Baylor College of Medicine, Houston, TX 77021, USA; manasa.kokonda@bcm.edu; 2 Center of Innovation in Quality, Effectiveness & Safety, Michael E DeBakey Veterans Association, Houston, TX 77021, USA; 3 South Central Mental Illness Research Education Clinical, VISN 16, Veteran Association, Houston, TX 77021, USA; 4 Department of Internal Medicine, Division of Pulmonary, Critical Care, & Sleep Medicine, University of Texas Medical Branch, Galveston, TX 77555, USA; risultan@utmb.edu (R.S.); bbduong@utmb.edu (B.B.D.); senagy@utmb.edu (S.E.N.); mfzaidan@utmb.edu (M.F.Z.); mibaig@utmb.edu (M.M.B.); justin.b.seashore@kp.org (J.S.); 5 School of Medicine, Baylor College of Medicine, Houston, TX 77030, USA; bryan.grigg@bcm.edu; 6 Department of Nutrition, Metabolism, & Rehabilitation Sciences, University of Texas Medical Branch, Galveston, TX 77555, USA; rachelrdeer@gmail.com; 7 Sealy Center on Aging, University of Texas Medical Branch, Galveston, TX 77555, USA

**Keywords:** sleep quality, depression, stress, anxiety, trauma, impact of events, long-COVID symptoms, post-acute sequelae of SARS-CoV-2 infection, PASC

## Abstract

A growing body of research documents the persistence of physical and neuropsychiatric symptoms following the resolution of acute COVID-19 infection. To the best of our knowledge, no published study has examined the interaction between insomnia and mental health. Accordingly, we proposed to examine new diagnoses of insomnia, and referrals to pulmonary and sleep medicine clinics for treatment of sleep disorders, in patients presenting to one post-acute COVID-19 recovery clinic. Additionally, we aimed to examine the relationship between poor sleep quality, depression, anxiety, and post-traumatic stress. Patients presented to the clinic on average 2 months following COVID-19 infection; 51.9% (*n* = 41) were hospitalized, 11.4% (*n =* 9) were in the intensive care unit, 2.5% (*n =* 2) were on a mechanical ventilator, and 38.0% (*n =* 30) were discharged on oxygen. The most commonly reported symptom was fatigue (88%, *n* = 70), with worse sleep following a COVID-19 infection reported in 50.6% (*n* = 40). The mean PSQI score was 9.7 (82.3%, *n* = 65 with poor sleep quality). The mean GAD-7 score was 8.3 (22.8%, *n =* 14 with severe depression). The mean PHQ-9 was 10.1 (17.8%, *n =* 18 with severe anxiety). The mean IES-6 was 2.1 (54.4%, *n =* 43 with post-traumatic stress). Poor sleep quality was significantly associated with increased severity of depression, anxiety, and post-traumatic stress. Future work should follow patients longitudinally to examine if sleep, fatigue, and mental health symptoms improve over time.

## 1. Introduction

A growing body of research documents the persistence of physical and neuropsychiatric symptoms following the resolution of an acute COVID-19 infection that has been termed long-COVID, or post-acute sequelae of SARS-CoV-2 infection (PASC) [1]. Post COVID-19 conditions occur in individuals with a history of probable or confirmed SARS CoV-2 infection, usually 3 months from the onset of COVID-19 with symptoms, and last for at least 2 months and cannot be explained by an alternative diagnosis [2]. Of all symptoms associated with long-COVID, sleep disturbances are among the most prevalent, affecting more than 4 out of 10 individuals in a recent meta-analysis [3]. Depression and anxiety are also frequently reported and may affect more than 3 in 10 individuals following the resolution of acute symptoms [4]. Poor sleep quality following an acute COVID-19 infection is associated with female gender, medical comorbidities, and indicators of disease severity including number of symptoms on admission, duration of hospital stay, necessity of mechanical ventilation, and hypertension requiring vasopressors [5,6,7,8]. Initial data suggest that sleep disorders may improve with increased time since recovery, but whether sleep quality returns to baseline has yet to be seen [9,10].

Disordered sleep is strongly associated with anxiety and depression, and it is estimated that two-thirds to three-fourths of patients with anxiety and depression report difficulties sleeping [11,12]. To what extent disordered sleep in long-COVID represents sequelae of affective disorders is unknown. Despite research to date examining neuropsychiatric symptoms and long-COVID, to the best of our knowledge, no published study has examined the interaction between insomnia and mental health. Accordingly, we proposed to examine new diagnoses of insomnia, and referrals to pulmonary and sleep medicine clinics for treatment of sleep disorders, in patients presenting to one post-acute COVID-19 recovery clinic. Additionally, we aimed to examine the relationship between poor sleep quality, depression, anxiety, and post-traumatic stress.

## 2. Materials and Methods

Patients (≥18 years old with positive COVID-19 PCR or antibody test) were referred, after hospital discharge or from the community, for follow-up care at a post-COVID-19 recovery clinic between August 2020 and September 2021. Every patient hospitalized at the University of Texas Medical Branch (UTMB) for COVID-19 was offered an appointment at the UTMB post-COVID-19 recovery clinic. The multi-specialty clinic evaluated all patients via a standard screening tool during either a 1 h long telehealth or in-person appointment. The screening battery included: an initial history, functional assessment, behavioral assessment, and nutritional assessment. In a subset of patients, a sleep assessment was also included. 

For this retrospective study, we examined sleep characteristics after a COVID-19 infection. Collected variables included: demographics (age, gender, race, ethnicity, comorbidities, prior sleep disorders); acute COVID-19 infection details; and COVID-19 clinic baseline visit details (generalized anxiety disorder (GAD-7), Patient Health Questionnaire (PHQ-9), and Impact of Events Scale (IES-6), to allow for evaluation of the presence of anxiety, depression, and post-traumatic stress symptoms, respectively). Sleep was assessed with the Pittsburgh Sleep Quality Index (PSQI). We also collected data on referrals for sleep testing and new-onset sleep disorder diagnosis. This study was approved by the University of Texas Medical Branch (UTMB) Institutional Review Board (IRB #20-0193). Validated measures of the study included:

Pittsburgh Sleep Quality Index (PSQI). The PSQI is a 19-item validated survey instrument designed to assess general sleep quality. Seven components include: (1) sleep duration, (2) sleep disturbance, (3) sleep latency, (4) daytime dysfunction due to sleepiness, (5) sleep efficiency, (6) overall sleep quality, and (7) sleep medication use. Each of the sleep components yields a score ranging from 0 to 3, with 3 indicating the greatest dysfunction. The sleep component scores are summed to yield a total score ranging from 0 to 21, with the higher total score (referred to as global score) indicating worse sleep quality. In distinguishing good and poor sleepers, a global PSQI score > 5 yields a sensitivity of 89.6%, and a specificity of 86.5%.

Patient Health Questionnaire-9 (PHQ-9). The PHQ-9 is a 9-item self-reported, diagnostic and severity measure for current (in the prior 14 days) depression, using criteria from the Diagnostic and Statistical Manual of Mental Disorders, fourth edition (DSM-IV). The 9 items include: (1) anhedonia, (2) depressed mood, (3) insomnia or hypersomnia, (4) fatigue or loss of energy, (5) appetite disturbance, (6) guilt or worthlessness, (7) diminished ability to think or concentrate, (8) psychomotor agitation or retardation, and (9) suicidal thoughts. Scores for each item range from 0 (“not at all”) to 3 (“nearly every day”). The PHQ-9 total score is the sum of scores for the 9 items for each participant, and ranges from 0 to 27. Among clinic patients, a score ≥ 10 is associated with a sensitivity of 88% and a specificity of 88% in diagnosing major depressive disorder (MDD).

Generalized Anxiety Disorder-7 (GAD-7). The GAD-7 is a 7-item questionnaire developed to identify probable cases of generalized anxiety disorder (GAD) and measure the severity of GAD symptoms. The GAD-7 items include: (1) nervousness, (2) inability to stop worrying, (3) excessive worry, (4) restlessness, (5) difficulty in relaxing, (6) easy irritation, and (7) fear of something awful happening. The GAD-7 asks participants to rate how often they have been bothered by each of these 7 core symptoms over the past 2 weeks. Scores for each item range from 0 (“not at all”) to 3 (“nearly every day”). The GAD-7 total score is the sum of scores for the 7 items for each participant, and ranges from 0 to 21. The GAD-7 has good reliability, factorial validity, and concurrent validity; the optimal cutoff score is 7, with a sensitivity of 73.3% and a specificity of 67.3%.

Impact of Events-6 (IES-6). The IES-6is a brief scale measuring the severity of distress following exposure to traumatic events, and is a useful screening instrument for research in epidemiological studies or in clinical practice, simplified by Thoresen on the basis of the Impact of Events Scale Revised (IES-R) and highly correlated to IES-R. IES-6is a 6-item self-report measure of psychological response to trauma, with each item rated on a scale from 0 to 4. Its 3 subscales (intrusion, avoidance, and hyperarousal) are closely affiliated with PTSD symptoms. It can be anchored to any specific event, such as the COVID-19 epidemic. The average score *S* of IES-6is categorized as follows: *S* < 1.09 = normal; 1.09 ≤ *S* < 1.5 = showing stress symptoms; *S* ≥ 1.5 = may be diagnosed with PTSD. 

### Statistical Analysis

Descriptive statistics were calculated for demographic variables, hospital variables, post-COVID-19 clinic symptoms, baseline ICD-9/10 variables, sleep quality (PSQI), and outcome measures, which were represented as mean, standard deviation (SD) for continuous variables, and frequency and proportion for categorical variables. Correlation analysis was performed between sleep quality (PSQI measures) and outcome variables (GAD-7, PHQ-9, IES-6). Correlation analysis was also performed between demographics, hospital variables, comorbidities, and baseline ICD-9/10 variables versus outcome variables. The variables that correlated with outcome variables at the significant *p* value of 0.05 level were considered for inclusion in the multiple regression model. Outcome variables were checked for normality and homogeneity tests to satisfy the distribution assumptions. A stepwise regression method was used to find the best fit regression model. As the outcome variables are continuous, linear regression was used. All tests were conducted two-sided, with a significance level of 0.05 and *p*-values were adjusted using the Bonferroni procedure when conducting multiple variables. All analyses were performed using SAS software version 9.4 (SAS Institute, Inc., Cary, NC, USA).

## 3. Results

*Demographics* (Table 1). Participants (*n =* 79) were on average 48.2 ± 12.4 years old with a BMI of 33.1 ± 8.5. The majority were female (69.6%, *n =* 55), white (86%, *n =* 68) and non-Hispanic (73.4%, *n =* 58). The average number of days to visit the clinic from COVID-19 positive was 62.01 ± 66.5. The changes in sleep due to COVID were reported as worse sleep after COVID-19 (50.6%, *n =* 40), no change (48.1%, *n =* 38), and better sleep after COVID-19 (1.3%, *n =* 1).

*Descriptive statistics* (Table 2 and Table 3). Descriptive statistics of sleep quality (PSQI measures) and outcomes measures (GAD-7, PHQ-9, IES-6) were calculated. These measures are categorized based on their cut-off scores. The severity levels of these measures were represented in frequency and percentage.

*Sleep disorders* (Table 4). We used ICD-9/10 codes for insomnia, hypersomnia, sleep apnea, and excessive daytime sleepiness to examine sleep disorder diagnosis in the electronic health records prior to, and following presentation to, the post-COVID-19 clinic. Patients with a history of sleep disorders before COVID-19 infection were categorized under pre-COVID-19. Patients with a new onset of sleep disorders with a diagnosis occurring after COVID-19 infection, along with patients with previous sleep disorder diagnosis, were categorized under post-COVID-19.

**Figure 1:**
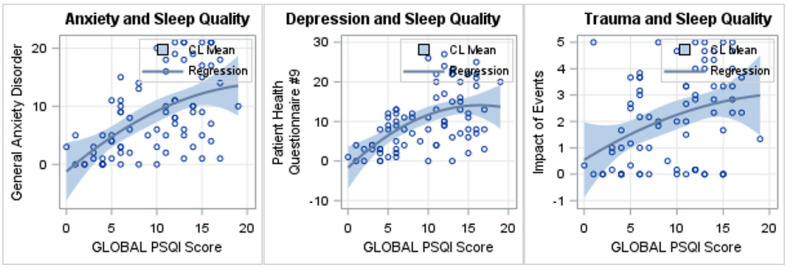
Linear regression plots between sleep quality (global PSQI) and outcomes.

*Sleep quality and Outcome measures* (Table 5 and Figure 1). Poor sleep quality (i.e., increases in PSQI scores) predicted greater anxiety severity (i.e., an increase in GAD-7 score). There is significant increase in anxiety scores with an increase in PSQI daytime dysfunction (*p* = 0.03), and with an increase in global PSQI (*p* < 0.0001).

Poor sleep quality (i.e., increases in PSQI scores) predicted greater depression severity (i.e., an increase in PHQ-9 score). There is significant increase in depression scores with an increase in PSQI daytime dysfunction due to sleep (*p* < 0.0001), PSQI sleep quality (*p* = 0.0001), and global PSQI (*p* < 0.0001).

Poor sleep quality (i.e., increases in PSQI scores) predicted greater traumatic stress (i.e., an increase in IES-6score). There is significant increase in traumatic stress symptoms with an increase in PSQI daytime dysfunction due to sleep (*p* = 0.0013), PSQI sleep quality (*p* = 0.02), and global PSQI (*p* = 0.0013).

Regression line depicts as follows:

(a) With an increase in global PSQI (poor sleep quality), there is a significant increase in general anxiety disorder-7 (GAD-7; worse anxiety), *p* < 0.0001;
(b) With an increase in global PSQI (poor sleep quality), there is a significant increase in Patient Health Questionnair-9 (PHQ-9; worse depression), *p* < 0.0001;
(c) With an increase in global PSQI (poor sleep quality), there is a significant increase in Impact of Events-6 (IES-6; worse trauma symptoms), *p* = 0.013.

## 4. Discussion

The present study evaluated the association between sleep quality and mental health symptoms (i.e., depression, anxiety, and post-traumatic stress) among patients that presented to a post-COVID-19 recovery clinic for long-term symptoms following a COVID-19 infection. Patients presented at the post-COVID-19 clinic an average of 62 days following COVID-19 infection. Of the 79 participants, 51.9% (*n =* 41) were hospitalized, 11.39% (*n =* 9) were in the intensive care unit, 2.53% (*n =* 2) were on a mechanical ventilator, and 37.97% (*n =* 30) were discharged on oxygen. The most commonly reported symptom when reporting to the post-COVID-19 clinic was fatigue, with 88% (*n =* 70) of the sample reporting fatigue. In addition, 50.6% (*n =* 40) reported their sleep worsened following COVID-19 infection. 

The mean PSQI score was 9.69, with 82.3% (*n =* 65) of the sample scoring at or above the clinical cut-off (≥5) for poor sleep quality. The mean GAD-7 score was 8.28, with 22.8% (*n =* 18) scoring in the severe anxiety range. The mean PHQ-9 was 10.10, with 17.8% (*n =* 14) scoring in the severe depression range. The mean IES-6was 2.09, with 54.4% (*n =* 43) scoring at or above the clinical cut-off (>1.5) for post-traumatic stress. When examining ICD-9/10 diagnosis in the electronic health record, we found 4 patients had a new diagnosis of insomnia, 10 sleep apnea, and 2 excessive daytime sleepiness since COVID-19 infection. When examining associations, we found that self-reported global sleep quality and daytime dysfunction were significantly associated with depression, anxiety, and post-traumatic stress symptoms. These results suggest that 50% (*n =* 39) of patients found sleep to be negatively impacted by COVID-19 infection, and that poor sleep quality persisted when presenting at the post-COVID-19 clinic (on average 2 months following infection). Patients mostly reported fatigue and daytime dysfunction as a residual symptom following COVID-19 infection. 

Our results are consistent with prior studies that found high rates of poor sleep, depression, and anxiety in patients who were infected with COVID-19. In addition, these symptoms appear to persist for longer than two months following infection. It remains unclear if these symptoms will resolve naturally with time. Further, there is emerging evidence that supports the idea that mood disorders lie on a spectrum, or a dimensional view [13]. There are several genetic and neurobiological studies lending support to the idea that these conditions are not discrete categories but rather, have common biological underpinnings, and may form at least part of a continuum or affective disorder spectrum. This has important implications for the diagnosis and treatment of mood in long-COVID and other patients.

A strength of our study is that we were able to examine baseline conditions and hospital variables by examining the medical record, in addition to relying on patient self-report. Few studies have examined the long-term effects of COVID-19 on sleep, fatigue, and mental health symptoms. 

There are several limitations to our study that should be noted when evaluating the results from this study. First, this was a sample of convenience of patients that presented to the long-COVID recovery clinic following COVID-19 infection. All patients were referred upon being discharged from the hospital. In addition, patients not hospitalized patients could be referred by their primary care provider. Thus, there may be bias related to which patients opted to schedule an appointment the UTMB post-COVID-19 recovery clinic. Secondly, sleep quality was limited to self-reporting. It would be useful to collect objective measures of sleep, such as actigraphy, that are not limited by cognitive issues that were specifically reported as ongoing symptoms, when presenting at the post-COVID-19 clinic, by 37% (*n =* 29) of the sample. In addition, we did not inquire with those with a history of sleep disorder diagnosis if insomnia or excessive daytime sleepiness was ongoing, or a new episode following COVID-19 infection. The cross-sectional and observational nature of our design meant that we cannot make any assertions about directionality or causality, including any causal role of COVID-19 infection on poor sleep quality, fatigue, depression, anxiety, or post-traumatic stress. Finally, the short-term follow up period limited our assessment of long-term symptoms or spontaneous remission rate. It should be noted that average time of symptoms was 2 months and was limited by the assessments in the clinic visit appointments, not the remittance of symptoms at 2 months. Study design made it difficult to determine length of symptom duration. 

Future work should address the limitations of the present study. To strengthen these findings, objective measures of sleep (actigraphy) should be used in future studies. Future longitudinal studies should continue to monitor sleep, fatigue, and mental health symptoms at multiple time points, to see if these symptoms remit spontaneously or are severe enough to require intervention. 

## 5. Conclusions

In summary, many patients who presented to a post-COVID-19 recovery clinic reported fatigue, poor sleep quality, and other symptoms. Patients presented to the clinic on average two months following COVID-19 infection and the symptoms had not remitted yet. A total of 4 patients received a new diagnosis of insomnia, 10 sleep apnea, and 2 excessive daytime sleepiness following COVID-19 infection. Poor sleep quality was significantly associated with increased severity of depression, anxiety, and post-traumatic stress. Future work should follow patients longitudinally to examine if sleep, fatigue, and mental health symptoms spontaneously remit with time. 

## Figures and Tables

**Table 1:** Demographics and hospitalization characteristics of study cohort (*n =* 79).

Variable	
Age; mean (SD)	48.2 (12.4)
BMI; mean (SD)	33.10 (8.5)
Days to clinic visit from COVID-19 positive	62.01 (66.51)
Gender; *n (%)*	
Female	55 (69.6)
Male	24 (30.4)
Race; *n (%)*	
Black or African American	9 (11.39)
White	68 (86.08)
American Indian/Alaskan Native	1 (1.27)
Asian	1 (1.27)
Ethnicity; *n (%)*	
Hispanic	21 (26.58)
Non-Hispanic	58 (73.42)
**Hospital variables**	
Hospitalized; *n (%)*	
Yes	41 (51.90)
No	38 (48.10)
Intensive care unit; *n (%)*	
Yes	9 (11.39)
No	70 (88.61)
Mechanical ventilation; *n (%)*	
Yes	2 (2.53)
No	77 (97.47)
High flow nasal canula; *n (%)*	
Yes	21 (26.58)
No	58 (73.42)
Nasal canula; *n (%)*	
Yes	36 (45.57)
No	43 (54.43)
Discharge on oxygen; *n (%)*	
Yes	30 (37.97)
No	49 (62.03)
**Post-COVID-19 Clinic Symptoms Reported**	
Weakness at clinical visit; *n (%)*	
Yes	57 (72.15)
No	22 (27.85)
Fatigue; *n (%)*	
Yes	70 (88.61)
No	9 (11.39)
Cognitive change; *n (%)*	
Yes	29 (36.71)
No	50 (63.29)
**Baseline ICD-9/10 Diagnosis**	
Atrial fibrillation; *n (%)*	
Yes	2 (2.53)
No	77 (97.47)
Diabetes mellitus; *n (%)*	
Yes	12 (15.19)
No	67 (84.81)
Hypertension; *n (%)*	
Yes	33 (41.77)
No	46 (58.23)
Coronary obstructive pulmonary disorder; *n (%)*	
Yes	3 (3.80)
No	76 (96.20)
Asthma; *n (%)*	
Yes	17 (21.52)
No	62 (78.48)
Chronic kidney disorder; *n (%)*	
Yes	3 (3.80)
No	76 (96.20)
Stroke; *n (%)*	
Yes	7 (8.86)
No	72 (91.14)
Congestive heart failure; *n (%)*	
Yes	2 (2.53)
No	77 (97.47)
Coronary artery disease; *n (%)*	
Yes	18 (22.78)
No	61 (77.22)
Cancer; *n (%)*	
Yes	5 (6.33)
No	74 (93.67)
Liver disease; *n (%)*	
Yes	4 (4.06)
No	75 (94.94)
**Self-reported sleep changes**	
Sleep changes due to COVID-19; *n (%)*	
Better after COVID-19	1 (1.27)
No change	38 (48.10)
Worse after COVID-19	40 (50.63)

Sample size = 79, values presented as mean (standard deviation) or *n (%)*, SD = standard deviation, BMI = body mass index.

**Table 2:** Descriptive statistics of sleep quality and outcome measures.

Variable	Mean (SD)
PSQI global	9.69 (4.8)
PSQI C1: sleep quality	1.78 (0.99)
PSQI C2: sleep latency	1.77 (1.12)
PSQI C3: sleep duration	1.07 (1.23)
PSQI C4: sleep efficiency	1.20 (1.27)
PSQI C5: sleep disturbance	1.37 (0.56)
PSQI C6: use of sleep medication	1.16 (1.34)
PSQI C7: daytime dysfunction	1.34 (0.92)
General anxiety disorder-7 (GAD-7)	8.28 (6.88)
Patient Health Questionnaire-9 (PHQ-9)	10.10 (7.41)
Impact of Events-6 (IES-6)	2.09 (1.74)
PSQI = Pittsburgh Sleep Quality Index	

**Table 3:** Clinical cut-off frequencies.

Severity Levels	Score	*n (%)*
Global PSQI		
Good sleep quality	<5	14 (17.7)
Poor sleep quality	≥5	65 (82.3)
IES-6		
No-mild stress symptoms	<1.75	36 (45.6)
Severe stress / PTSD	≥1.75	43 (54.4)
PHQ-9		
Euthymic	0–4	24 (30.4)
Mild depression	5–9	18 (22.8)
Moderate depression	10–14	17 (21.5)
Moderate to severe depression	15–19	6 (7.5)
Severe depression	20–27	14 (17.8)
GAD-7		
Minimal anxiety	0–4	29 (36.7)
Mild anxiety	5–9	20 (25.3)
Moderate anxiety	10–14	12 (15.2)
Severe anxiety	15–21	18 (22.8)

Acronyms: PSQI = Pittsburgh Sleep Quality Index. IES = Impact of Events-scale 6. PHQ-9 = Physical Health Questionnaire-scale 9. GAD-7 = General Anxiety Disorder-scale 7. PTSD = Post-traumatic Stress Disorder.

**Table 4:** Descriptive statistics of sleep disorders between pre-COVID-19 and post-COVID-19 infection.

Sleep Disorders	Pre-COVID-19 *n* (%)	Post-COVID-19 *n* (%)
Insomnia	13 (16.5)	17 (21.5)
Hypersomnia	1 (1.3)	1 (1.3)
Sleep apnea	21 (26.6)	31 (39.2)
Excessive daytime sleepiness	3 (3.8)	5 (6.3)

NOTE: Post-COVID-19 represents patients with NEW onset of sleep disorders after COVID-19 infection, along with patients who had history of sleep disorders before COVID-19 infection.

**Table 5:** Regression table between Sleep quality and outcome variables.

	GAD-7	PHQ-9	IES-6
PSQI global	0.65 (0.15) *	0.82 (0.15) *	0.13 (0.03) *
PSQI C1: sleep quality	1.65 (1.03)	2.85 (0.69) *	0.45 (0.19) *
PSQI C2: sleep latency	0.67 (0.69)	0.73 (0.68)	−0.12 (0.19)
PSQI C3: sleep duration	0.16 (0.95)	−0.76 (0.93)	−0.15 (0.26)
PSQI C4: sleep efficiency	0.24 (0.86)	1.16 (0.84)	0.18 (0.24)
PSQI C5: sleep disturbance	2.10 (1.41)	3.16 (1.38)	−0.01 (0.38)
PSQI C6: use of sleep meds	0.38 (0.49)	−0.45 (0.49)	−0.04 (0.14)
PSQI C7: daytime dysfunction	1.76 (0.79) *	3.28 (0.76) *	0.76 (0.22) *

* Indicates significant at alpha 0.05 level. Values are represented as β (SE). β = regression coefficient (represents the slope of the linear relation of the predictor variable and the outcome variable), SE = standard error. Acronyms: PSQI = Pittsburgh Sleep Quality Index.

## Data Availability

The data presented in this study are available on request from the corresponding author. The data are not publicly available due to privacy protection reasons.
